# Editorial: Peripheral Nervous System-Machine Interfaces (PNS-MI)

**DOI:** 10.3389/fnbot.2017.00054

**Published:** 2017-10-24

**Authors:** Michael Wininger, Panagiotis Artemiadis, Claudio Castellini, Patrick Pilarski

**Affiliations:** ^1^Prosthetics and Orthotics Program, University of Hartford, West Hartford, CT, United States; ^2^Cooperative Studies Program, United States Department of Veterans Affairs, West Haven, CT, United States; ^3^Department of Biostatistics, Yale University, New Haven, CT, United States; ^4^Department of Mechanical and Aerospace Engineering, Arizona State University, Tempe, AZ, United States; ^5^Robotics and Mechatronics Center, German Aerospace Center (DLR), Oberpfaffenhofen, Germany; ^6^Department of Medicine, University of Alberta, Edmonton, AB, Canada

**Keywords:** abandonment, control, database, detection, electromyography, myoelectric, prosthetics, society

**Editorial on the Research Topic**

**Peripheral Nervous System-Machine Interfaces**

## Introduction

The Peripheral Nervous System-Machine Interfaces (PNS-MI) Workgroup is now in its fifth year of activity, and with this Editorial completes its fourth annual deliverable. Following our first Workshop at International Conference on Rehabilitation Robotics (ICORR)-2013 in Seattle, and its Proceedings document (Castellini et al.), we convened at ICORR-2015 in Singapore, and for 2016, cast a wide net for contributions to this Research Topic, seeking both to summarize the discussions in Singapore and also to gather new perspectives.

## About the Research Topic (RT)

In our advertisement for this RT, we solicited contributions that either (a) advanced the frontier of prosthetic technology or (b) bridged the gap between the laboratory and the clinic. We are most delighted to be able to present ten articles written by 55 authors at 27 different institutions (representing universities, hospitals, clinics, government, and industry) from Europe, Asia, North- and South America. We Topic Editors believe that the impact of this RT may be best described as advancing the frontier in three domains: debut of novel technologies, new approaches to reducing device abandonment, and under-served or exciting new patient populations.

The new technologies introduced here extend the Workgroup’s long-standing interest in EMG-based prosthetic control: utterly novel and potentially transformative detection paradigms (Höppner et al.); comprehensive testing of convolutional neural networks with simplistic architecture versus classical classification approaches to a large hand movement database (Atzori et al.); and introduction of a novel, wireless wearable biosensor for measuring force myography and electromyography simultaneously (Connan et al.).

The PNS-MI emphasis on reducing device rejection is a recent addition to our research thrusts; this RT brings together an important distinction between classifier algorithms and clinical scores/ADL (Vujaklija et al.); finding a differential EMG-to-position mapping that ensures highest coherence with hand movements for naturalistic and intuitive control (Fani et al.); a work that coins the concept of the prosthetic homolog, and tests its putative criticality for acceptance and embodiment (Dornfeld et al.); and a new method for assessing user functionality in myoelectric control signal of upper-limb prostheses with application to in-clinic, in-lab, and also real-world (Chadwell et al.).

Starting with the Workshop in Singapore, the Workgroup expanded its coverage to include a wider range of patient populations and prosthetic applications. This RT expands this initiative through a hybridized EMG-mechanotechnology viz. the control of an augmentative hand prosthesis (Hussain et al.) and a classification of hand motions from different wrist positions among partial-hand amputation patients (Adewuyi et al.). And while historically our group has been interested primarily in upper-limb prosthetics, we were delighted to publish a work on vibrotactile feedback system for the control of transtibial prosthesis (Chen et al.).

## Next Steps: 2017 Activity

In our fifth year, we shall cycle back to Workshops. We will next convene at the 2017 American Orthotics and Prosthetics Association (AOPA) World Congress in Las Vegas (Symposium on Multi-Scale Integration in Upper-Limb Prosthetics, Saturday, September 9). Coincident with AOPA’s 100th Anniversary, we find it fitting to bring the work of our group to the AOPA World Congress, where thousands of practitioners, researchers, manufacturers, physicians, and facility owners gather from around the globe to learn, share, and collaborate.

## Next Steps: Workgroup Evolution

Our Workshop at AOPA will mark our debut as the formative Society for Prosthetics. As our group evolves its portfolio of research interests and efforts to foster collaborative exchange, we believe that it is only natural to self-identify in the tradition of academic associations that have served other sectors of medicine and engineering so well over time.

The PNS-MI Workgroup was a terrific launching pad, and accomplished its main goal perfectly: it served as the assembly point for prosthetics researchers from across the globe, to identify common objectives, opportunities for further research, and to discuss their progress with each other. But our Group wants to engage with our process partners: patients, clinical providers, payers, and students, as well as researchers from allied fields. As an informal Workgroup, we are inherently limited in our visibility and appeal to these niches; as a society, offer a much broader platform of offerings to the community.

Following our third Workshop at AOPA-2017, we will pursue a third proceedings document in 2018, wherein we shall aspire to accomplish: (1) continued dissemination of our valuable prosthetics research and (2) roadmap for the Society. We thank our many dedicated PNS-MI pioneers, and look forward to ever-higher impact through the Society for Prosthetics.

## Author Contributions

The four authors contributed equally to editing the Research Topic.

## Conflict of Interest Statement

The authors declare that the research was conducted in the absence of any commercial or financial relationships that could be construed as a potential conflict of interest.

